# Genistein Induces Deleterious Effects during Its Acute Exposure in Swiss Mice

**DOI:** 10.1155/2014/619617

**Published:** 2014-05-22

**Authors:** Prabhat Singh, Sharad Sharma, Srikanta Kumar Rath

**Affiliations:** ^1^Department of Biological Sciences, Indian Institute of Science Education and Research (IISER), Bhopal, Madhya Pradesh 462023, India; ^2^Toxicology Division, CSIR-Central Drug Research Institute, Lucknow, Uttar Pradesh 226031, India

## Abstract

Genistein is a soy derived isoflavone. It has wide variety of therapeutic effects against certain diseases including cancer. Although toxic effects of genistein have been studied, its effect on the gene expression and the reason behind toxicity have not been identified yet. In the present study, genistein was administered to age and body weight matched Swiss mice at the doses of 125, 250, 500 and 1000 mg/kg. The biomarkers of hepatotoxicity in serum, liver histology, oxidative stress parameters in tissue homogenates, and global gene expression were examined. Elevated alanine aminotransferase (ALT), aspartate aminotransferase (AST), and alkaline phosphatase (ALP) levels and degenerated liver tissue were observed in 500, and 1000 mg/kg genistein treated groups. Oxidative stress was significant at these doses as considerable increase in lipid peroxidation (LPO) and decrease in total glutathione (GSH) were observed. Gene expression analysis showed 40 differentially expressed genes at twofold change and *P* < 0.05. Differentially expressed genes were corresponding to different biologically relevant pathways including metabolic and oxidative stress pathways. In 500 mg/kg group, Cyp4a14, Sult1e1, Gadd45g, Cidec, Mycs, and so forth genes were upregulated. These results suggested that the higher dose of genistein can produce several undesirable effects by affecting multiple cellular pathways.

## 1. Introduction

Genistein is a major soy isoflavone which occurs naturally in diet. A wide variety of soy derived food products contain an ample amount of genistein. Genistein has numerous beneficial effects like bone health improvement [1], antilipogenic [2], antitumor [3], antioxidant [4], anticarcinogenic [5], and estrogenic [6]. Recent evidence suggested that genistein is a potential candidate for cancer chemotherapy [7]. In the USA, the average daily dietary intake of isoflavones is 1.1–1.3 mg/day while it varies between 10 and 110 mg/day in China and Japan [8]. Due to high production of soy based foodstuffs in Asia, Asian population is incessantly exposed to isoflavones. Indeed, potential chemopreventive effects amplified the soy consumption. Despite having useful therapeutic properties, genistein is receiving attention as a major environmental contaminant on the basis of increasing conventional acute, subchronic, and chronic safety studies in various animal models [9]. Genistein exerts adverse effects on reproductive system of different rodent models [10] and elevates the relative uterine weight and downregulates the progesterone receptor in uterine epithelia [11]. Several* in vitro* studies reported its clastogenic and mutagenic potential [12–14]. Genistein induces chromosomal breakage [15] and micronucleus [16] formation in different cell lines. It affects cell growth and proliferation [17]. It induces apoptosis in nerve cells at high doses through intracellular calcium ion release and p42/44 mitogen-activated protein kinase [18]. Genistein is capable of transforming cells [19] which lead to different kinds of cancer during developmental stage of animals [20–22].

Although effects of genistein in different* in vitro* and* in vivo* models have been studied, early induced gene biomarker and the reason behind the toxicity have not been identified yet. In the present study, oligonucleotide microarray has been employed for a better understanding of gene expression signatures. In addition, effect of genistein on antioxidant status of liver was assessed in dose-dependent manner. Acute doses of genistein (125, 250, 500, and 1000 mg/kg) were administered in Swiss albino mice through intraperitoneal route. Doses and route of exposure of genistein were based on the reported literature [7]. After considering the body surface area index [23], doses were selected which were also corresponding to the human exposure of isoflavonoids [8].

## 2. Materials and Methods

### 2.1. Animals and Drug Administration

25–30 gm, 10–12-week-old male Swiss albino mice (*Mus musculus*) were obtained from Laboratory Animal Division, Central Drug Research Institute, Lucknow. Animals were randomly allocated to the following groups:

 Group I: vehicle treated animals (control),
 Group II: genistein 125 mg/kg treated,
 Group III: genistein 250 mg/kg treated,
 Group IV: genistein 500 mg/kg treated,
 Group V: genistein 1000 mg/kg treated.

Each group contains six animals. All animal procedures were performed in compliance with institutional animal ethics guidelines (113/09/Toxicol./IAEC dated 10.7.09). Animals were acclimatized to optimal conditions of temperature (25 ± 2°C) and light/dark cycle (12 hr each) before drug administration. Genistein was dissolved in DMSO and intraperitoneally (IP) administered to Swiss albino mice. DMSO treated animals were used as control. Food and water were supplied* ad libitum*. Animals were sacrificed after 24 hr of treatment.

### 2.2. Chemicals and Biochemicals

Genistein (5,7-dihydroxy-3-(4-hydroxyphenyl)-4H-1-benzopyran-4-one, CAS number 446-72-0), serum bovine albumin, HEPES, EDTA, EGTA, DTT, PMSF, protease inhibitor cocktail, Ponceau S stain, and Bradford reagent were procured from Sigma, St. Louis, USA. DMSO, KCl, MgCl_2_, and H_2_O_2_ were purchased from Merck, India. ALT, AST, and ALP estimation kits were purchased from Beckman Coulter, Ireland. TRIzol, First-Strand Synthesis Supermix for qRT-PCR and SYBR GreenER qPCR Supermix Universal kit were purchased from Invitrogen, California, USA. Primary antibodies such as rabbit polyclonal IgG SOD1, actin, and secondary antibody goat anti-rabbit IgG-HRP were procured from Santa Cruz Biotechnology, California. Purified mouse anti-Hsp70 antibody was purchased from BD Biosciences, USA. Enhanced chemiluminescent (ECL) kit was procured from GE Healthcare, UK.

### 2.3. Blood Collection and Serum Biochemistry

During autopsy blood was withdrawn from each animal and allowed to stand undisturbed for 30 min. The serum was separated and levels of ALT, AST, and ALP were estimated using an automated biochemical analyzer (Beckman Coulter, California, USA).

### 2.4. Liver Tissue Processing and Histology

A part of liver tissue was snap-frozen in liquid N_2_ within 2 min of sacrifice and subsequently stored at −80°C for further use in RNA and protein isolation and the second part was frozen for different enzymatic assays. Another part of tissue was fixed in 10% formal saline for histological investigations. Fixed liver tissues were washed overnight, dehydrated through graded alcohols, and embedded in paraffin wax. Serial sections of 5 *μ*m thickness were stained with hematoxylin and eosin (H and E) for histological examinations.

### 2.5. Liver Tissue Biochemistry

Liver tissue homogenates were used for antioxidant enzymatic assays. Malondialdehyde (MDA) concentration (a measure of lipid peroxidation, LPO), total glutathione content (GSH), and antioxidant enzymes activities (superoxide dismutase, catalase, glutathione peroxidase, and glutathione reductase) were estimated using standard tests [24–29]. Lowry method was used to estimate the total protein content with serum bovine albumin as a standard [30]. Readings of all assays were taken in spectrophotometer (Powerwave XS, BIO-TEK, USA).

### 2.6. RNA Isolation, cDNA Labeling, and Hybridization

50 mg frozen liver tissue was crushed in liquid N_2_ and immediately homogenized (Heidolph, Germany) in 1 ml TRIzol to isolate total RNA. After quantification from spectrophotometer (Amersham Biosciences, UK) and running on formaldehyde gel, RNA samples with approximately 2 : 1 ratio of 28S : 18S rRNA and 260/280 values ≥ 1.8 were used for gene expression analysis. Equal amounts of RNA from individuals of the same group were pooled to eliminate interindividual variations. RNA samples were labeled using the T7 promoter based-linear amplification to generate labeled complementary RNA (Agilent Quick-Amp Labeling Kit). Quality control (QC) was performed using nanodrop and cRNA was purified using Qiagen's RNeasy minikit. The amplified and labeled cRNA was hybridized to mouse 60 K whole genome arrays using Agilent's* In Situ* Hybridization kit (Agilent microarray services, Genotypic Technology, Bangalore, India).

### 2.7. Scanning and Microarray Data Analysis

The arrays were washed with buffers and subsequently scanned with confocal laser scanner to collect raw data. Intensity values were extracted and percentile shift normalization was performed by using GeneSpring GX 11.0 software. It is a global normalization, where the locations of all spot intensities in an array are adjusted. This normalization takes each column in an experiment independently and computes the percentile of expression values for this array, across all spots (where *n* has a range from 0 to 100 and *n* = 75 is the median). It subtracts this value from the expression value of each entity. Analysis was done with respect to control samples and statistically significant difference between control and genistein administered mice was deduced with two sample* t*-tests and probes with *P* < 0.05 and twofold differential expression at all doses were identified. Raw and log-transformed data have been submitted to Gene Expression Omnibus database (http://www.ncbi.nlm.nih.gov/geo/query/acc.cgi?acc=GSE23523) and conform to MIAME guidelines developed by Microarray Gene Expression Data (MGED) Society.

### 2.8. Clustering Algorithm and Pathway Analysis

Clustering algorithm was applied for the identification of patterns in gene expression data. Hierarchical clustering was used to join data in a single group having the most similar expression profiles. Average linkage method was used to measure the pair-wise distance between entities in two clusters and application of Pearson uncentered correlation measured the similarity or difference between entities. Significant pathways for the differentially regulated genes were obtained using Biointerpreter (Genotypic Technology, Bangalore, India). Pathways that show *P* < 0.05 for differentially regulated genes were taken into consideration.

### 2.9. Quantitative Real Time PCR Analysis

Real time PCR analysis was performed according to the supplier protocol (Invitrogen, California, USA) using Superscript III First-Strand Synthesis Supermix for qRT-PCR and SYBR GreenER qPCR Supermix Universal kit in 20 *μ*L volumes per well of 96-well clear optical reaction plates. The components of reaction were SYBR Green PCR Master Mix (Invitrogen, California, USA), cDNA template, forward and reverse primers (Table 1), and nuclease-free water (Sigma, USA). PCR reactions were performed in Light Cycler 480 Real Time PCR instrument and analyzed according to accompanying software instructions (Roche Diagnostics Ltd., Switzerland). Beta-actin was used as an internal control and used to normalize ratios between samples. For primer pair, melting curve analysis was performed according to the instrument software instructions. Program was an initial incubation of 50°C for 2 min hold (UDG incubation) and 95°C for 10 min followed by 40 cycles at 95°C for 15 s, 60°C for 60 s. Relative change in mRNA level between control and treated groups were calculated by using 2^−ΔΔ*C*_T_^ method.

### 2.10. Western Blot Analysis

Proteins were isolated from liver tissue using modified protocol [31]. Tissues from control and treatment groups were homogenized with 5–10 volumes of lysis buffer (200 mM HEPES, 10 mM KCl, 1.5 mM MgCl_2_, 1 mM EDTA, 1 mM EGTA, 1 mM DTT, 2 mM PMSF, and 1X protease inhibitor cocktail). Cellular debris was spun down at 20,000 g for 30 min at 4°C and supernatants were used as whole protein extract. Isolated proteins were quantified using Bradford reagent. 50 *μ*g protein from each sample was separated on 15% SDS-PAGE and transferred on to a nitrocellulose membrane using a semidry electroblotting apparatus (GE Healthcare, UK). Transfer was examined by Ponceau S stain and washed with triple distilled water until the stain disappeared. Membrane was overnight blocked in 5% nonfat dried milk at 4°C. Blocking membrane was washed with 0.1% PBST and probed with primary antibodies (actin, SOD1, and Hsp70). After primary antibody incubation further washing was done in 0.1% PBST. Membrane was incubated in HRP conjugated secondary antibody and washed again. Enhanced chemiluminescent detection system was used to develop the blots. Blots were further used for densitometric analysis and normalization.

### 2.11. Statistical Analysis

Data were expressed as mean ± standard error of means (SEM). Group means were compared by one-way analysis of variance (ANOVA) followed by Newman-Keuls multiple comparison test. The differences in data obtained were considered statistically significant when *P* < 0.05.

## 3. Results

### 3.1. Level of Serum Biomarkers

**Figure 1:**
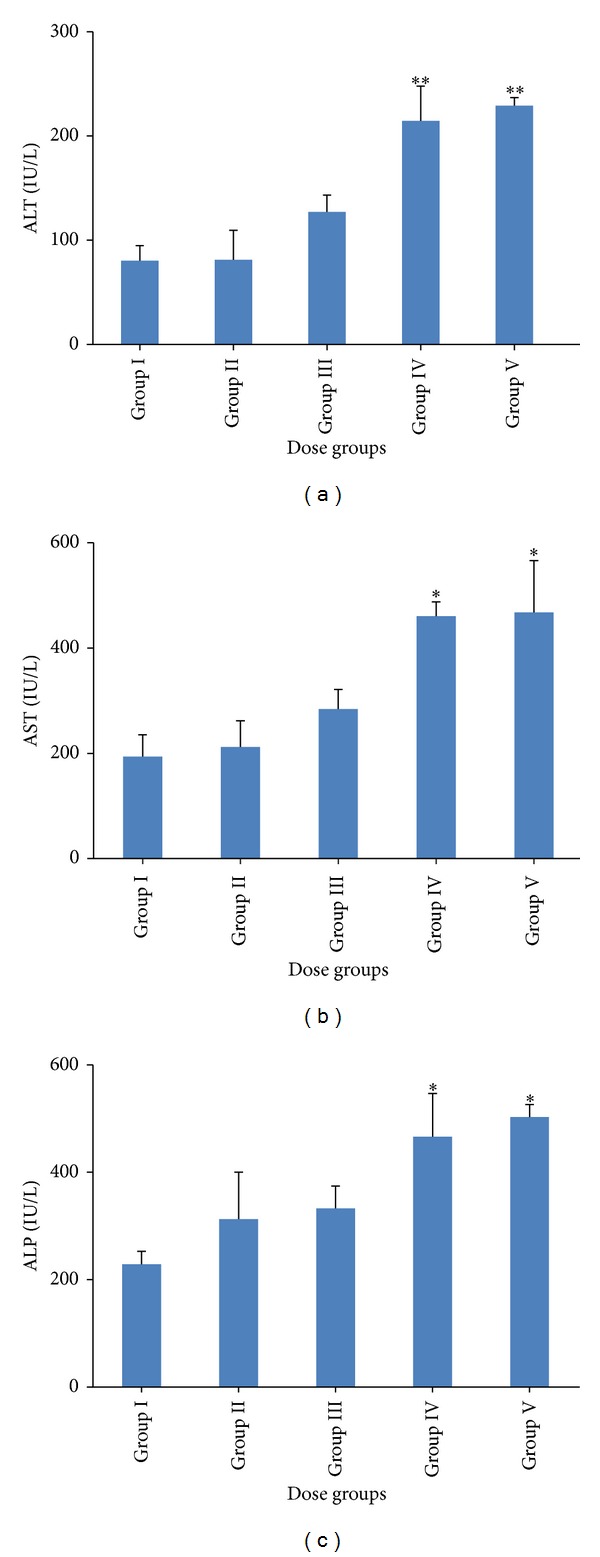
Level of serum biomarkers of hepatotoxicity: bar diagrams are showing the levels of (a) alanine amino transferase (ALT), (b) aspartate amino transferase (AST), and (c) alkaline phosphatase (ALP) following the administration of genistein at different doses (i.e., Group I: vehicle treated control, Group II: 125 mg/kg, Group III: 250 mg/kg, Group IV: 500 mg/kg, and Group V: 1000 mg/kg). The asterisks indicate the significance of differences (*P < 0.05, **P < 0.01, and ***P < 0.001) in comparison to control.

A significant increase in serum ALT (*P* < 0.01), AST (*P* < 0.05), and ALP (*P* < 0.05) level was found in higher doses (Groups IV and V) of genistein as compared to control (Group I). However, serum ALT, AST, and ALP level did not change in Group II and III animals as compared to control (Figures 1(a), 1(b), and 1(c)).

### 3.2. Histological Examination of Liver Tissue

**Figure 2:**
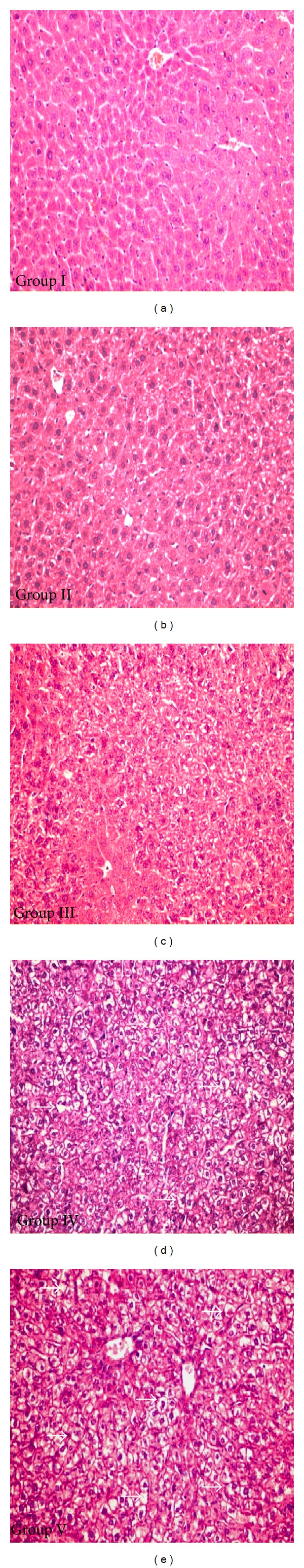
Histological examination of liver sections: photomicrograph of transverse sections of mice liver. (a) Group I: vehicle treated control, (b) Group II: 125 mg/kg, (c) Group III: 250 mg/kg, (d) Group IV: 500 mg/kg, and (e) Group V: 1000 mg/kg. Hydropic changes and degeneration of cytoplasm within hepatocytes were observed in Group IV 500 mg/kg and Group V 1000 mg/kg genistein treated animals. Arrows indicate the degenerated hepatocytes in 500 and 1000 mg/kg genistein treated groups.

Liver sections of Group II and Group III animals showed well distributed normal hepatocytes, central vein, bile duct, and hepatic artery with no histological alterations as compared to control (Group I). In the liver sections of Group IV and Group V animals, hydropic changes were observed in hepatocytes (Figures 2(a), 2(b), 2(c), 2(d), and 2(e)); these changes were characterized by ballooning and degeneration.

### 3.3. Lipid Peroxidation Level

**Figure 3:**
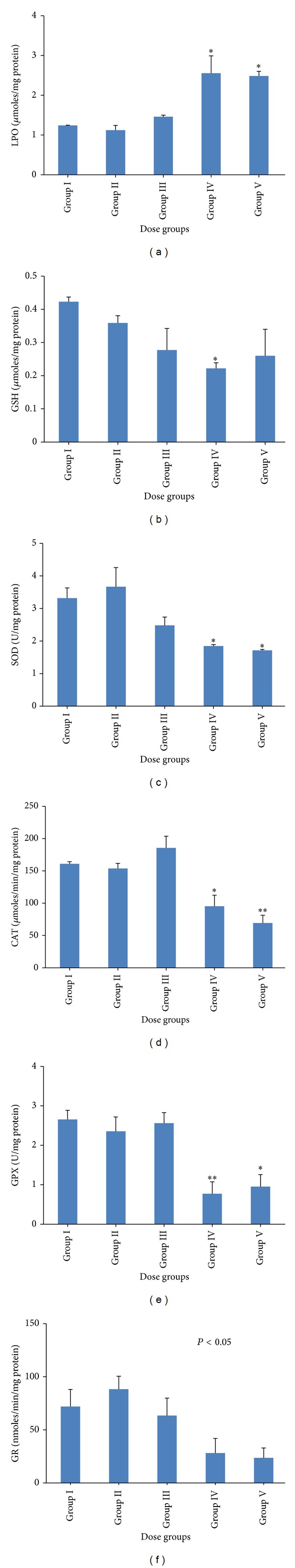
Level of oxidative stress parameters: bar diagrams are showing (a) lipid peroxidation level, (b) total glutathione content, (c) superoxide dismutase (SOD) activity, (d) catalase (CAT) activity, (e) glutathione peroxidase (GPX) activity, and (f) glutathione reductase (GR) activity in mice liver following the administration of genistein at different doses (i.e., Group I: vehicle treated control, Group II: 125 mg/kg, Group III: 250 mg/kg, Group IV: 500 mg/kg, and Group V: 1000 mg/kg).

Malondialdehyde (MDA), a secondary product of lipid peroxidation, was not altered in Group II and III animals as compared to control (Group I). However, a significant increase in MDA concentration was observed in the liver of Group IV and V (*P* < 0.05) genistein treated animals (Figure 3(a)) as compared to control.

### 3.4. Total Glutathione Estimation

Total glutathione content in higher treatment groups (Group IV; *P* < 0.05 and Group V) was significantly decreased as compared to control (Group I). In other dose groups (Groups II and III), glutathione content did not change as compared to control (Figure 3(b)).

### 3.5. Activity, mRNA, and Protein Level of SOD

**Figure 4:**
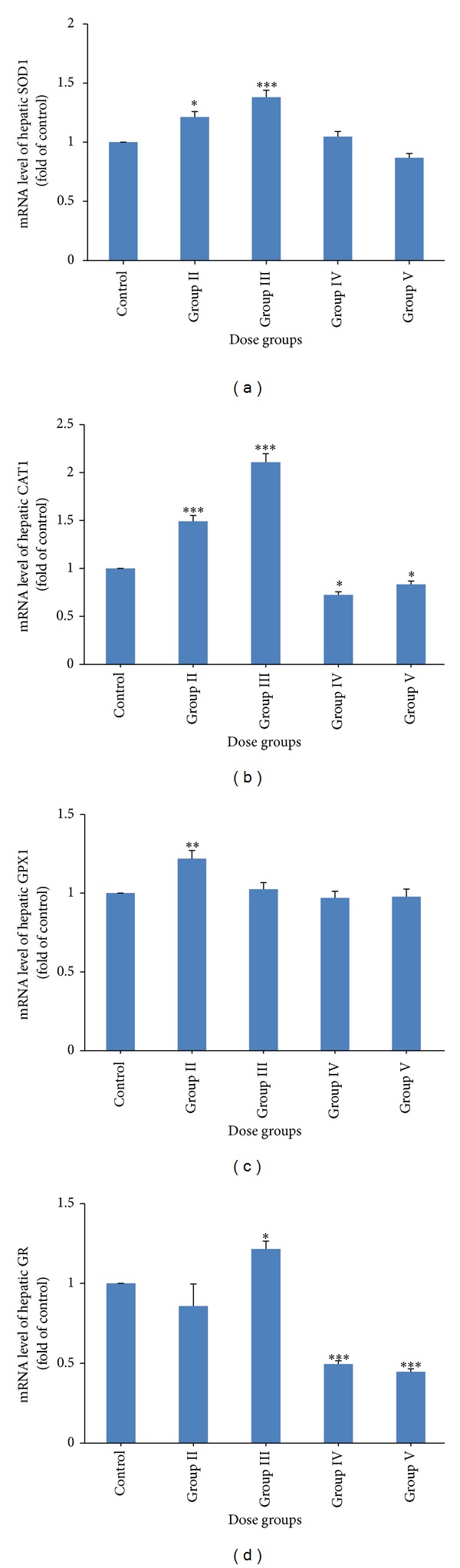
Quantitative real time PCR analysis of stress regulated genes: (a) SOD1, (b) CAT1, (c) GPX1, and (d) GR genes in the mice liver treated with different doses of genistein (control, 125, 250, 500, and 1000 mg/kg). Bars: I: control, II: 125 mg/kg, III: 250 mg/kg, IV: 500 mg/kg, and V: 1000 mg/kg.

**Figure 5:**
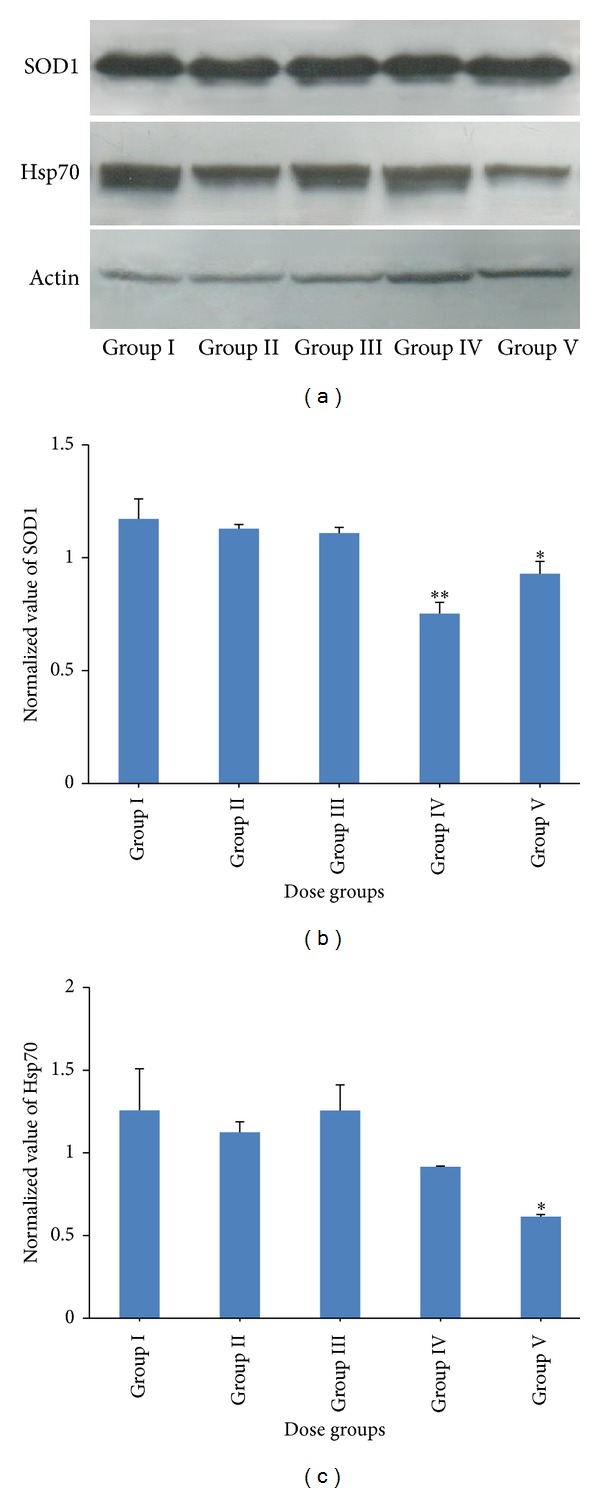
Western blot analysis of SOD1 and Hsp70. (a) Western blots of SOD1, Hsp70, and actin proteins. (b, c) Bar diagrams are showing the relative band intensity of (b) SOD1 and (c) Hsp70 after normalization with actin. Bars: I: vehicle treated control, II: 125 mg/kg, III: 250 mg/kg, IV: 500 mg/kg, and V: 1000 mg/kg.

In Group IV and V genistein treated animals, SOD activity (Figure 3(c); *P* < 0.05) was decreased significantly; however, no significant changes were observed in Group II and III genistein treated animals as compared to control group. SOD1 protein level (Figures 5(a) and 5(b)) was decreased in Group IV and V animals as compared to control; however, no significant change was observed in Group II and III animals. In lower treatment groups (Groups II and III), SOD1 mRNA (Figure 4(a)) level was increased; however, in the highest treatment group (Group V) mRNA level was decreased. In Group IV animals, SOD1 mRNA level did not change as compared to control (Group I).

### 3.6. Activities and mRNA Level of CAT, GPX, and GR

In higher dose groups of genistein (Groups IV and V), CAT and GPX activities (Figures 3(d) and 3(e)) were significantly decreased. CAT and GPX activities did not alter in lower treatment groups (Groups I and II) of genistein as compared to control. A decrease in GR activity (Figure 3(f)) was found in Group IV and V animals. In lower treatment groups (Groups I and II), GR activity did not change as compared to control.

In Group IV and V animals, a significant decrease in CAT1 and GR mRNA level was found; however, GPX1 mRNA did not change in these groups as compared to control (Figures 4(b), 4(c), and 4(d)). CAT1 mRNA level was significantly increased in lower treatment groups (Groups II and III) (Figure 4(b)). GPX1 mRNA level was increased in Group II animals; however, it did not change in Group III animals as compared to control (Figure 4(c)). GR mRNA level did not change in Group II animals; however, it was increased in Group III animals (Figure 4(d)).

### 3.7. Protein Level of Hsp70

Protein level of Hsp70 was decreased during the genistein treatment (Figure 5(a)). Densitometry analysis and normalization with actin showed the maximum decrease in Group IV and V (*P* < 0.05) genistein treated groups as compared to control (Figure 5(c)).

### 3.8. Differential Gene Expression Analysis

Following genistein exposure, mRNA expression in mice liver was assessed with 60,000 unique probes. A statistical stringent criterion (twofold change and *P* < 0.05) identified 40 differentially expressed probes consisting of 20 upregulated and 20 downregulated probes (Table 2). Few differentially expressed probes have no sequence similarity with any known gene and have not been assigned any biological function. Moreover, real time PCR analysis of selected genes (Table 1) showed similar trend of regulation as found in differential expression to microarray results.

### 3.9. Affected Pathways and Cluster Analysis

**Figure 6:**
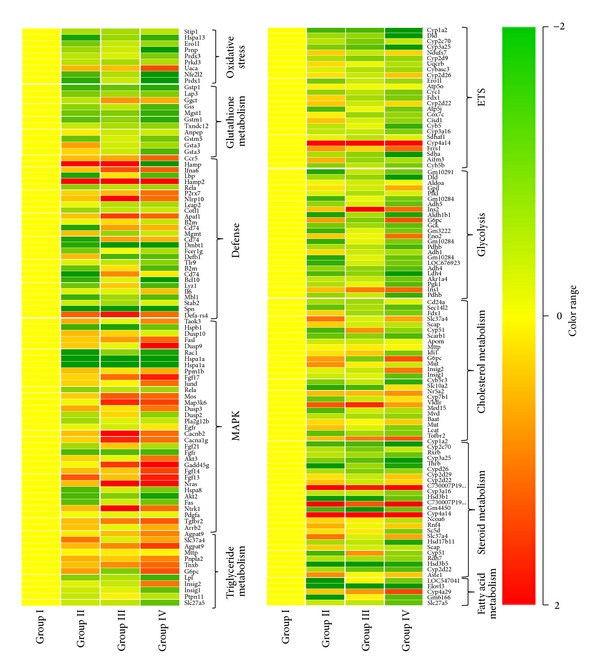
Cluster analysis of differentially expressed genes: a portion of the* k*-means clusters of differentially regulated genes (oxidative stress, glutathione metabolism, defense, MAPK, triglyceride, ETS, glycolysis, cholesterol, steroid, and fatty acid related genes) following the administration of genistein at different doses 125 mg/kg (Group II), 250 mg/kg (Group III), and 500 mg/kg (Group IV) involved in different pathways as compared to vehicle treated control (Group I).

**Figure 7:**
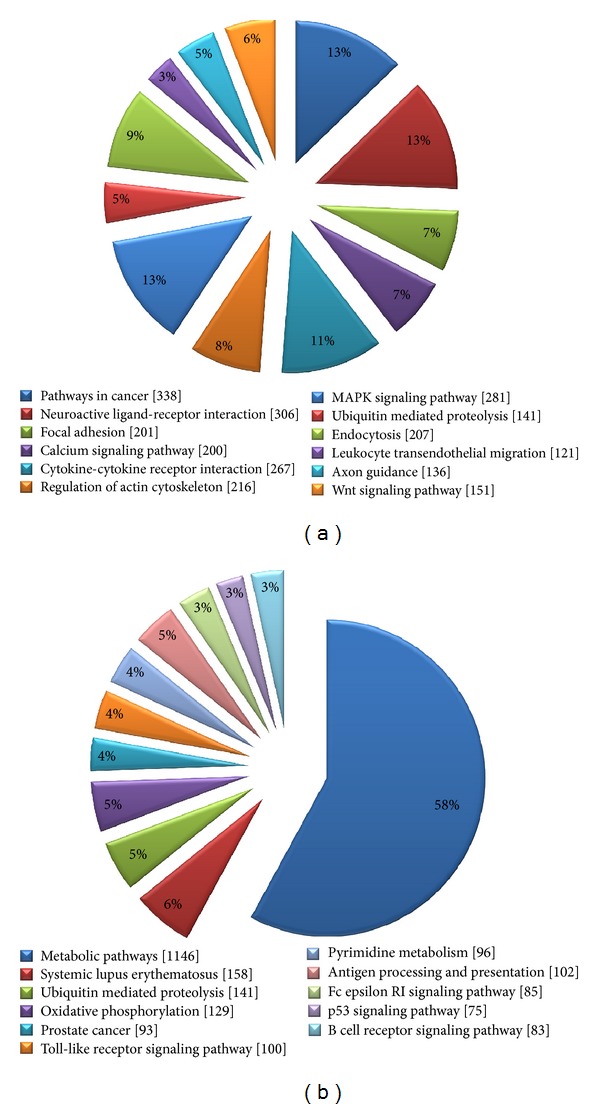
Pathway analysis: pie chart is showing the significant pathways of (a) upregulated and (b) downregulated genes obtained after using Biointerpreter software in 500 mg/kg genistein (Group IV) treated animals. Pathways were identified after applying the criterion of *P* < 0.05. Number within the square bracket [] specifies the number of genes in the pathway in genome.

Differentially regulated genes were clustered using hierarchical clustering to identify significant gene expression patterns. The most similar expression profiles are joined together to form a group. These were further joined in a tree structure, until all data forms a single group. Genes were classified based on functional category and pathways using Gene Spring GX Software and Genotypic Biointerpreter-Biological Analysis Software. Major pathways were identified that showed signs of genistein induced perturbations including oxidative stress, metabolic, inflammatory, and MAPK kinase related pathways (Figure 6; see Table S1 in supplementary material available online at http://dx.doi.org/10.1155/2014/619617). We further analyzed the differentially regulated genes of Group IV (500 mg/kg genistein) animals to detect the changes at transcript level and found that a large number of genes related to cancer pathways were upregulated; however, the metabolic pathway related genes were majorly downregulated (Figure 7).

## 4. Discussion

In the present study, effects of genistein on the mice liver were evaluated. Acute doses of genistein were intraperitoneally administered. We observed hepatotoxicity in 500 and 1000 mg/kg genistein treated animals in terms of elevated serum ALT, AST, and ALP level. These three parameters (ALT, AST, and ALP) are established hepatotoxicity biomarkers and utilized to monitor acute liver injury [32]. Few other flavonoids have already been reported to induce significant hepatic damage during intraperitoneal exposure. Galati et al. found the 4-fold increased plasma ALT level after 24 hr when tea polyphenols like EGCG (120 mg/kg), propyl gallate (170 mg/kg), gallic acid (500 mg/kg), and tannic acid (120 mg/kg) were administered intraperitoneally in CD-1 mice [33]. In our previous study [34], acute doses of apigenin (100 and 200 mg/kg) elevated the serum biomarkers level (ALT, AST, and ALP) in Swiss albino mice. In the present study, we observed the histological alterations which were characterized by ballooning and degeneration of hepatocytes in higher doses (500 and 1000 mg/kg) further confirm the hepatotoxicity at these doses. Unaffected liver histoarchitecture in 125 and 250 mg/kg doses supported the serum findings evocative of nontoxic effects of genistein at these doses. This may be due to the acute treatment of genistein. Previous investigators also accounted for negative correlation between altered serum markers and histological changes [32].

By knowing the fact that the flavonoids may act as prooxidant and may generate oxidative stress which might be the reason for hepatotoxicity, we examined the activity and expression of major antioxidant enzymes. One of the consequences of oxidative stress is the elevation in LPO level. In our study, a significant increase in LPO at higher doses (500 and 1000 mg/kg) rendered a clear indication of ROS generation. Other flavonoids have also been reported to increase LPO level during ROS generation [35]. In the present study, decreased activity and expression of SOD, CAT, GPX, and GR in higher treatment groups (500 and 1000 mg/kg) might be the consequence of decreased* de novo* synthesis of enzymes [36] or irreversible inactivation of enzymes from increased free radical generation [37] through genistein metabolism. Decrease in GR along with GSH suggested the overall reduction in GSH/GSSG ratio which may shift the biological system towards different biological states like proliferation, differentiation, apoptosis, or necrosis [38]. Furthermore, the dose-dependent reduction in Hsp70 expression by genistein indicated the apoptosis induction [39]. It has been reported that the Hsp synthesis is blocked by quercetin (a flavonoid) [40].

Microarray analysis was performed for 125, 250, and 500 mg/kg genistein treated animals to find out the earlier changes in gene biomarkers before the onset of the liver injury. Genomic markers are more reliable in short term acute toxicity studies where the phenotypic signs and symptoms may have not been fully developed [41]. In the present study, 40 differentially regulated genes were identified at twofold change and *P* < 0.05. To obtain the profoundness of gene expression results, we grouped genes under selected functional categories: (i) stress and glutathione metabolism related genes, (ii) defense and MAPK pathway related genes, (iii) ETS and glycolysis pathway related genes, and (iv) fatty acid, cholesterol, steroid, and triglyceride metabolism related genes.

*(i) Stress and Glutathione Metabolism Related Genes.* The most striking finding of our study is the massive downregulation of oxidative stress and glutathione metabolism related genes. For example, Nuclear Factor (Erythroid-Derived 2)-Like 2 (NRF2), Glutathione S-transferase pi 1 (GSTP1), Microsomal glutathione S-transferase 1 (MGST1) were down regulated. Expression of different isoforms of peroxiredoxins, for example, PRDX3, was also decreased. PRDX3 is located in mitochondria and guards emergent tumor cells against apoptosis [42]. It is reported that the absence or low level of PRDX3 declines the ability of mitochondria to neutralize ROS and potentiates early apoptosis in MCF7 cells when exposed to PP2 (a derivative of pyrimidine) [43]. In the present study, its downregulation indicated the induction of apoptosis in mice liver cells. Moreover, we found the decreased mRNA expression of Prnp, Stip1, Hsp70, and Sod genes. Stip1 provides potential to germ cells to survive in stress conditions [44] and exist in a macromolecular complex with the proteins of Hsp70 and 90 families [45]. Prnp, a cellular prion protein, cooperates with Stip1 and regulates superoxide dismutase activity in neuronal cell lines [46]. Coordinated downregulation of these genes suggested the augmentation of stress within liver cells. Furthermore, downregulation of glutathione synthetase gene which is involved in GSH biosynthesis corroborated the finding of GSH depletion in higher doses of genistein.

*(ii) Defense and MAPK Pathway Related Genes.* Genistein modulated the expression of defense and MAPK related pathway genes. Administration of 500 mg/kg genistein induced the transcriptional upregulation of certain immunomodulatory genes, for example, interferons (Ifna6), interleukins (Il6), and chemokines receptor (Ccr5) genes. In previous reports, IL-6 was upregulated in intravesical bacillus Calmette-Guerin (BCG) therapy of superficial bladder cancer [47] and IFN-*α*6 expression was increased with other subtypes of interferon alpha (IFN-*α*2, IFN-*α*4, IFN-*α*5, IFN-*α*7, and IFN-*α*10) in HIV-1 patients at different stages [48]. Similarly, CCR5 expression was increased in adult rats infected with Borna disease virus (BDV) [49]. Chemokines and their receptors participate in many pathophysiologic conditions, such as inflammation and autoimmunity. In the present study, upregulation of P2RX7 indicated the regulation of immune function and neurotransmitter release by genistein. P2RX7 is a purinergic ATP-binding calcium channel expressed in microglial cells and considered a candidate gene in type I diabetes [50]. The upregulation of fibroblast growth factor 17 (FGF17) and epidermal growth factor receptor (EGFR) by genistein clearly indicated the stimulation of different signaling pathways which have been known to be involved in cancer. FGF17 either enhances cellular proliferation or inhibits apoptosis [51] and EGFR induces signaling pathway in different kinds of cancer, namely, lung and anal cancer [52]. We found the downregulation of apoptosis-inducing factor (AIF) which pointed towards apoptosis induction. AIF is a caspase-independent apoptosis effector and protects neurons from oxidative stress-induced apoptosis* in vivo* [53].

*(iii) ETS and Glycolysis Pathway Related Genes.* mRNA of genes involved in ETS and glycolysis pathways were differentially expressed by genistein treatment. Cyp4a14 was induced more than 4.4-fold in 500 mg/kg genistein treated animals. Cyp4a14 is a member of cytochrome family CYP450 and plays a key role in metabolism of endogenic substances and xenobiotics [54]. It is reported that these proteins may interact with flavonoids by three ways: flavonoids can induce biosynthesis of certain CYPs, flavonoids can modulate enzymatic activity of CYPs, and flavonoids can be metabolized by several CYPs [54]. During metabolism, cytochrome P450 produces other metabolites having biological activities unlike from parent compound [55]. Thus, it can be speculated that genistein metabolites rather than the parent compound might have mediated the biological response. We found that the succinate dehydrogenase complex, subunit A (SDH A) was downregulated more than twofold in 500 mg/kg genistein dose group. SDH A accepts electrons from succinate during the conversion of succinate to fumarate in citric acid cycle. Its downregulation clearly indicated the impairment in metabolic regulation at a high dose of genistein. In the present study, differential regulation of other genes like Ndufs7, Cyc1, and Cyb5 of electron transport chain might have induced the premature electron leakage which ensued in oxidative stress.

*(iv) Fatty Acid, Cholesterol, Steroid, and Triglyceride Metabolism Related Genes.* It is well recognized that CYP450 genes do not only participate in xenobiotic metabolism but are also involved in fatty acid, cholesterol, steroid, and triglyceride metabolism. In the present study, differential expression of CYP450 family genes like Cyp4a29, Cyp7b1, Cyp4a14, Cyp2d10, Cyp2d26, Cyp7b1, Cyp3a25, Cyp2d9, and Cyp3a41b by different doses of genistein suggested the comparable regulation mechanism by CYP enzymes that might be involved in oxidative stress related metabolic pathways. We found the differential expression in solute carrier family genes (Slc27a5, Slc10a2, and Slc37a4) which have been reported to be involved in vacuole formation in hepatocytes through transport of fatty acids [56]. Lower doses of genistein were not toxic; however, higher doses potentially induced the hepatocellular vacuolization. In the present study, upregulation of Sult1e1, a phase 2 metabolism gene, indicated the possibility of sulfation of genistein. This might have contributed the resistance against this compound and conferred the glutathione depletion by enhancing the alternative route of genistein detoxification. Moreover, genistein modulated the steroid, fatty acid, and triglyceride metabolic pathways by regulating the mRNA expression of different enzymes (Hsd3b1, Hsd3b7, Hsd17b4, Hsd3b5, and Hsd17b11) and receptors (RXR-*α*).

In conclusion, elevated level of traditional serum biomarkers, degenerated hepatocytes, altered oxidative stress parameters, and differentially regulated genes are apparent indication of hepatotoxicity in genistein (500 and 1000 mg/kg) treated animals. Upregulation of cancer related pathways indicated the genistein induced perturbation which may lead to the several deleterious effects. This evidence suggested that there is a need to limit and regulate the dose of genistein in dietary supplements and in cancer therapeutics.

## Supplementary Material

Reply- Table S1. List of differentially expressed genes under selected functional categories. Genes were grouped under functional categories to obtain the profoundness of gene expression results. (i) Stress and glutathione metabolism related genes, (ii) Defense and MAPK pathway related genes, (iii) ETS and glycolysis pathway related genes, (iv) Fatty acid, cholesterol, steroid and triglyceride metabolism related genes.

## Figures and Tables

**Table 1:** List of primers used in quantitative real time polymerase chain reaction.

Gene	Forward primer (5′-3′)	Reverse primer (5′-3′)
*Actb *	GGCTGTATTCCCCTCCATCG	CCAGTTGGTAACAATGCCATGT
*Sod1 *	TTTTTGCGCGGTCCTTTCCTG	GGTTCACCGCTTGCCTTCTGCT
*Cat1 *	AGCGACCAGATGAAGCAGTG	TCCGCTCTCTGTCAAAGTGTG
*Gpx1 *	ATGTCGCGTCTCTCTGAGG	CCGAACTGATTGCACGGGAA
*Gr *	GGCAACAGGGTGATGATCTTC	CTGGAAAGTTCGGTCACATCC
*Sult3a1 *	AGATGTGGTAGGAAGCCTTTGG	CTTGTCTCACAACAGCATCCA
*Cyp4a14 *	GTCTCTCGGGGAGCAATATACG	ACCAATCCAGGGAGCAAAGAA
*H2-t24 *	TCGGCAATACTACAACAGCTCT	ATCGTAGCCATACTGCCAATG
*Adrbk2 *	AGGAGGGTTTGGGGAAGTTTA	CATGATCCTCTCGTTCAAAGCC
*Elovl3 *	TTCTCACGCGGGTTAAAAATGG	GAGCAACAGATAGACGACCAC
*Olfr1274 *	GTTCCTGCTTACGATGATGGC	GCAAGGGAATGGACAAAACCT
*Spn *	AACCATCAAATGTAGCCAGTGAC	GGTCTCGTTAGAGCTTGTTGTC
*Gemin6 *	GCCAACATTGTCCTCGTAAACT	TGTGGTCCCCTTCACTTATGG

**Table 2:** List of differentially expressed genes identified at *P* < 0.05 and twofold change.

Gene symbol	Regulation	Group II	Group III	Group IV	Gene ontology
*Sult3a1 *	Up	5.58	7.64	8.27	Sulfotransferase activity, transferase activity, amine sulfotransferase activity
*Hamp2 *	Up	5.08	3.51	2.05	Killing of cells of another organism, defense response to bacterium, cellular iron ion homeostasis, extracellular region, hormone activity, defense response to fungus
*Cyp4a14 *	Up	4.30	2.20	4.41	Oxidoreductase activity, metal ion binding, heme binding, alkane 1-monooxygenase activity, electron carrier activity, monooxygenase activity
*Extl3 *	Up	2.79	3.55	2.53	Transferase and glucuronyl-galactosyl-proteoglycan 4-alpha-N-acetylglucosaminyltransferase activity, metal ion binding, intrinsic to endoplasmic reticulum membrane, manganese ion binding
*Prei4 *	Up	2.36	2.19	3.99	Catalytic and hydrolase activity, glycerol-phosphodiester phosphodiesterase activity, phosphoric diester hydrolase activity, lipid, carbohydrate and glycerol metabolic process
*H2-T24 *	Up	2.04	3.09	2.10	Molecular function, cellular component, biological process, integral to membrane
*Adrbk2 *	Up	2.08	3.55	2.44	Protein amino acid phosphorylation, beta-adrenergic receptor kinase activity, signal transducer activity, G-protein coupled receptor and protein serine/threonine kinase activity
*Gm4976 *	Up	2.10	3.38	2.85	Hydrolase activity
*Mup20 *	Down	−3.43	−2.50	−4.58	Pheromone binding
*Hspa1a *	Down	−3.97	−4.56	−3.73	Response to stress, nucleotide binding, response to heat, cytoplasmic part, DNA repair, telomere maintenance, ATP binding
*Reg3b *	Down	−6.02	−2.84	−2.94	Acute-phase response, inflammatory response, sugar binding
*Rps6kb1 *	Down	−10.12	−9.44	−10.73	Protein amino acid phosphorylation, protein kinase B signaling cascade, transferase activity and kinase activity, germ cell development
*Olfr1274 *	Down	−10.80	−10.91	−10.32	Olfactory receptor activity, integral to membrane, sensory perception of smell, signal transducer activity, G-protein coupled receptor activity
*Spn *	Down	−10.55	−10.14	−9.85	Defense response to bacterium, integral to membrane, T cell costimulation, positive regulation of T cell proliferation, negative regulation of T cell proliferation, positive regulation of tumor necrosis factor biosynthetic process
*Elovl3 *	Down	−2.66	−2.91	−3.30	Endoplasmic reticulum, fatty acid and lipid biosynthetic process, integral to membrane
*Gemin6 *	Down	−11.48	−10.75	−11.28	Spliceosomal complex, RNA splicing, protein binding, mRNA processing

## Abbreviations

ALT: Alanine amino transferase
AST: Aspartate amino transferase
ALP: Alkaline phosphatase
ROS: Reactive oxygen species
LPO: Lipid peroxidation
GSH: Reduced glutathione
GSSG: Oxidized glutathione
PP2: 4-Amino-5-(4-chlorophenyl)-7-(t-butyl) pyrazolo [3,4-d] pyrimidine
IP: Intraperitoneal
MIAME: Minimum Information About a Microarray Experiment
MGED: Microarray Gene Expression Data
ETS: Electron transport system.
