# Supplementary material for: Genistein Induces Deleterious Effects during Its Acute Exposure in Swiss Mice
Source: Biomed Res Int. 2014 May 22;2014:619617. doi: 10.1155/2014/619617 (PMC4055018; doi:10.1155/2014/619617)
Supplement: Supplementary file 1 — Reply- Table S1. List of differentially expressed genes under selected functional categories. Genes were grouped under functional categories to obtain the profoundness of gene expression results. (i) Stress and glutathione metabolism related genes, (ii) Defense and MAPK pathway related genes, (iii) ETS and glycolysis pathway related genes, (iv) Fatty acid, cholesterol, steroid and triglyceride metabolism related genes. [file 619617.f1.pdf]

Table S1. List of differentially expressed genes under selected functional categories

Probe name	Gene symbol	Name	Fold geomean		
			Group II	Group III	Group IV
**Expression of oxidative stress and glutathione metabolism related genes**					
GT_Mm_44k_51_P100828	Prdx3	Peroxiredoxin 3	-0.497	-0.269	-0.984
GT_Mm_44k_51_P106397	Prkd3	Protein kinase D3	-0.548	-0.685	-0.619
GT_Mm_44k_51_P353183	Prdx1	Peroxiredoxin 1	-1.641	-0.405	-2.390
GT_Mm_44k_51_P386880	Stip1	Stress-induced pho`sphoprotein 1	-0.640	-0.678	-0.533
GT_Mm_44k_51_P411271	Nfe2l2	Nuclear factor, erythroid derived 2, like 2	-1.393	-0.607	-2.060
GT_Mm_44k_51_P437336	Uaca	Uveal autoantigen with coiled-coil domains and ankyrin repeats	0.587	0.506	1.418
GT_Mm_44k_51_P483280	Prnp	Prion protein	-0.740	-0.373	-1.998
GT_Mm_44k_52_P376804	Hspa13	Heat shock protein 70 family, member 13	-1.796	-0.771	-1.582
GT_Mm_44k_52_P423424	Ero1l	ERO1-like (S. cerevisiae)	-1.045	-0.432	-0.770
GT_Mm_44k_51_P231184	Anpep	Alanyl (membrane) aminopeptidase	0.148	-0.387	-0.377
GT_Mm_44k_51_P260169	Gstm5	Glutathione S-transferase, mu 5	-1.155	-0.307	-1.100
GT_Mm_44k_51_P287810	Mgst1	Microsomal glutathione S-transferase 1	-1.133	-0.987	-1.582
GT_Mm_44k_51_P289881	Txndc12	Thioredoxin domain containing 12	-0.436	-0.632	-0.845
GT_Mm_44k_51_P374464	Gstp1	Glutathione S-transferase, pi 1	-1.513	-1.170	-1.670
GT_Mm_44k_52_P358860	Gss	Glutathione synthetase	-0.856	-0.461	-1.391
GT_Mm_44k_52_P379256	Lap3	Leucine aminopeptidase 3	-1.002	-0.697	-0.967
GT_Mm_44k_52_P415215	Gstm1	Glutathione S-transferase, mu 1	-1.286	-0.868	-1.666
GT_Mm_44k_52_P432124	Gsta3	Glutathione S-transferase, alpha 3	0.329	-0.406	-1.382
GT_Mm_44k_52_P641758	Ggct	Gamma-glutamyl cyclotransferase	-0.516	0.848	0.657
**Expression of defense and MAPK related genes**					

GT_Mm_44k_00046	Defa-rs4	Defensin, alpha,, related sequence 4	1.262	1.845	1.173
GT_Mm_44k_01450	Mgmt	O-6-methylguanine-DNA methyltransferase	0.506	-0.826	-0.425
GT_Mm_44k_01532	Cd74	CD74 antigen	-1.725	0.747	0.604
GT_Mm_44k_02048	Ifna6	Interferon alpha 6	0.453	1.391	1.272
GT_Mm_44k_51_P117881	Leap2	Liver-expressed antimicrobial peptide 2	-0.137	-0.576	-0.662
GT_Mm_44k_51_P129012	B2m	Beta-2 microglobulin	-1.170	0.227	-1.353
GT_Mm_44k_51_P209527	Bcl10	B-cell leukemia/lymphoma 10	-1.547	-0.235	-2.186
GT_Mm_44k_51_P217218	Il6	Interleukin 6	-0.230	-0.231	0.501
GT_Mm_44k_51_P302576	Spn	Sialophorin	-10.559	-10.148	-9.855
GT_Mm_44k_51_P373619	Mbl1	Mannose-binding lectin (protein A) 1	-1.542	-0.727	-1.305
GT_Mm_44k_51_P385993	Rela	V-rel reticuloendotheliosis viral oncogene homolog A (avian)	-0.883	-0.532	-0.759
GT_Mm_44k_51_P402846	Stab2	Stabilin 2	-0.365	-0.682	-0.234
GT_Mm_44k_51_P405476	Fcer1g	Fc receptor, IgE, high affinity I, gamma polypeptide	-1.022	-0.077	-0.661
GT_Mm_44k_51_P432563	Tlr9	Toll-like receptor 9	-0.228	-0.486	-0.905
GT_Mm_44k_51_P454008	Lbp	Lipopolysaccharide binding protein	-1.750	0.227	-1.356
GT_Mm_44k_51_P500676	Dmbt1	Deleted in malignant brain tumors 1	-1.697	-3.165	-2.026
GT_Mm_44k_51_P506733	P2rx7	Purinergic receptor P2X, ligand-gated ion channel, 7	0.359	0.533	1.150
GT_Mm_44k_52_P21486	Hamp2	Hepcidin antimicrobial peptide 2	5.089	3.513	2.057
GT_Mm_44k_52_P321140	Defb1	Defensin beta 1	0.548	-1.418	-1.300
GT_Mm_44k_52_P435356	Cotl1	Coactosin-like 1 (Dictyostelium)	-0.798	-0.476	-0.434
GT_Mm_44k_52_P578732	Ccr5	Chemokine (C-C motif) receptor 5	0.502	0.670	1.258
GT_Mm_44k_52_P582059	Lyz1	Lysozyme 1	-1.130	0.413	-1.403

GT_Mm_44k_52_P590625	Nlrp10	NLR family, pyrin domain containing 10	0.467	2.532	1.162
GT_Mm_44k_52_P621991	Apaf1	Apoptotic peptidase activating factor 1	0.476	1.530	1.234
GT_Mm_44k_52_P669922	Hamp	Hepcidin antimicrobial peptide	2.966	3.175	-2.870
GT_Mm_44k_01225	Ppm1b	Protein phosphatase 1B, magnesium dependent, beta isoform	0.504	0.240	0.632
GT_Mm_44k_01846	Arrb2	Arrestin, beta 2	0.474	-0.162	0.644
GT_Mm_44k_51_P104418	Dusp10	Dual specificity phosphatase 10	0.533	0.350	-0.256
GT_Mm_44k_51_P117952	Hspa1a	Heat shock protein 1A	-3.974	-4.569	-3.734
GT_Mm_44k_51_P170371	Hspa8	Heat shock protein 8	-1.398	-0.302	-1.022
GT_Mm_44k_51_P170807	Map3k6	Mitogen-activated protein kinase kinase kinase 6	-0.099	1.708	1.501
GT_Mm_44k_51_P184385	Pla2g12b	Phospholipase A2, group XIIB	-0.732	-0.246	-0.446
GT_Mm_44k_51_P274223	Fgf17	Fibroblast growth factor 17	0.345	1.167	1.784
GT_Mm_44k_51_P305003	Ntrk1	Neurotrophic tyrosine kinase, receptor, type 1	0.507	2.811	1.170
GT_Mm_44k_51_P315904	Gadd45g	Growth arrest and DNA-damage-inducible 45 gamma	-0.065	1.572	2.718
GT_Mm_44k_51_P318488	Akt3	Thymoma viral proto-oncogene 3	0.358	-0.165	1.145
GT_Mm_44k_51_P345393	Fas	Fas (TNF receptor superfamily member 6)	-1.263	-0.337	-1.117
GT_Mm_44k_51_P357744	Jund	Jun proto-oncogene related gene d	0.347	0.086	1.160
GT_Mm_44k_51_P370090	Pdgfa	Platelet derived growth factor, alpha	-0.074	-0.184	-0.172
GT_Mm_44k_51_P385993	Rela	V-rel reticuloendotheliosis viral oncogene homolog A (avian)	-0.883	-0.532	-0.759
GT_Mm_44k_51_P396273	Nras	Neuroblastoma ras oncogene	0.531	3.918	1.947
GT_Mm_44k_51_P461748	Dusp3	Dual specificity phosphatase 3	0.675	0.205	1.080
GT_Mm_44k_51_P513	Rac1	RAS-related C3	-1.950	-0.878	-1.787

254		botulinum substrate 1			
GT_Mm_44k_52_P106259	Egfr	Epidermal growth factor receptor	-0.143	-0.338	0.178
GT_Mm_44k_52_P166755	Taok3	TAO kinase 3	0.652	-0.186	0.944
GT_Mm_44k_52_P168549	Fgf14	Fibroblast growth factor 14	0.528	0.449	1.492
GT_Mm_44k_52_P209514	Cacnb2	Calcium channel, voltage-dependent, beta 2 subunit	0.498	2.137	1.171
GT_Mm_44k_52_P235347	Fgf21	Fibroblast growth factor 21	-0.719	0.440	-0.624
GT_Mm_44k_52_P251403	Tgfbr2	Transforming growth factor, beta receptor II	0.513	1.025	1.321
GT_Mm_44k_52_P267243	Fgf13	Fibroblast growth factor 13	1.338	0.488	1.789
GT_Mm_44k_52_P2977	Akt2	Thymoma viral proto-oncogene 2	-1.394	-0.974	-1.834
GT_Mm_44k_52_P399934	Dusp2	Dual specificity phosphatase 2	-0.637	0.317	-0.885
GT_Mm_44k_52_P404533	Hspb1	Heat shock protein 1	-1.509	-1.775	-1.141
GT_Mm_44k_52_P42332	Mos	Moloney sarcoma oncogene	0.391	1.301	1.225
GT_Mm_44k_52_P611610	Cacna1g	Calcium channel, voltage-dependent, T type, alpha 1G subunit	0.353	1.725	1.148
GT_Mm_44k_52_P648601	Dusp9	Dual specificity phosphatase 9	0.417	-0.151	2.419
GT_Mm_44k_52_P77106	Fasl	Fas ligand (TNF superfamily, member 6)	0.416	0.785	1.211
**Expression of ETS and glycolysis related genes**					
GT_Mm_44k_00911	Cyp2d10,1,2	Cytochrome P450, family 2, subfamily d, polypeptide 22	-0.660	-1.147	-1.279
GT_Mm_44k_01406	Cyp4a14	Cytochrome P450, family 4, subfamily a, polypeptide 14	4.307	2.209	4.419
GT_Mm_44k_01473	Frrs1	Ferric-chelate reductase 1	0.793	0.303	1.262
GT_Mm_44k_01762	Ndufs7	NADH dehydrogenase (ubiquinone) Fe-S protein 7	0.385	-0.418	0.333
GT_Mm_44k_01942	Aifm3	Apoptosis-inducing factor, mitochondrion-associated 3	0.601	-0.809	-0.637
GT_Mm_44k_51_P100	Atp5j	ATP synthase, H+	-1.356	-0.125	-1.061

866		transporting, mitochondrial F0 complex, subunit F			
GT_Mm_44k_51_P120295	Cyp2d26	Cytochrome P450, family 2, subfamily d, polypeptide 26	0.234	-0.419	0.446
GT_Mm_44k_51_P124126	Cyp2d10,11,d22	Cytochrome P450, family 2, subfamily d, polypeptide 22	0.429	-0.409	0.300
GT_Mm_44k_51_P131800	Cyba	Cytochrome b-245, alpha polypeptide	-1.616	-0.678	-1.112
GT_Mm_44k_51_P134142	Cyp2c70	Cytochrome P450, family 2, subfamily c, polypeptide 70	-0.542	-1.009	-0.435
GT_Mm_44k_51_P138152	Cisd1	CDGSH iron sulfur domain 1	0.466	-0.295	-0.828
GT_Mm_44k_51_P141123	Cox5b	Cytochrome c oxidase, subunit Vb	0.324	-0.206	0.284
GT_Mm_44k_51_P160744	Ndufb3	NADH dehydrogenase (ubiquinone) 1 beta subcomplex 3	0.384	-0.183	-0.430
GT_Mm_44k_51_P168708	Atp5f1	ATP synthase, H+ transporting, mitochondrial F0 complex, subunit b, isoform 1	0.282	-0.469	-0.926
GT_Mm_44k_51_P177552	Ndufb9	NADH dehydrogenase (ubiquinone) 1 beta subcomplex, 9	0.398	-0.330	0.413
GT_Mm_44k_51_P183292	Atp5g1	ATP synthase, H+ transporting, mitochondrial F0 complex, subunit c (subunit 9), isoform 1	-0.520	-0.421	-0.767
GT_Mm_44k_51_P184284	Dld	Dihydrolipoamide dehydrogenase	-0.731	-0.804	-2.104
GT_Mm_44k_51_P263756	Ndufa7	NADH dehydrogenase (ubiquinone) 1 alpha subcomplex, 7 (B14.5a)	-0.166	0.061	-0.605
GT_Mm_44k_51_P291129	Fdx1	Ferredoxin 1	0.359	-0.700	-0.994
GT_Mm_44k_51_P295610	Cyc1	Cytochrome c-1	-0.857	-0.529	-1.025
GT_Mm_44k_51_P301289	Atp5k	ATP synthase, H+ transporting, mitochondrial F1F0	0.600	-0.399	0.219

		complex, subunit e			
GT_Mm_44k_51_P311434	Cyb5	Cytochrome b-5	-0.509	-0.162	-1.565
GT_Mm_44k_51_P324871	Cybasc3	Cytochrome b, ascorbate dependent 3	-0.275	-0.587	-0.731
GT_Mm_44k_51_P336827	Cyb5b	Cytochrome b5 type B	-0.910	-0.484	-1.036
GT_Mm_44k_51_P338728	Sdhaf1	Succinate dehydrogenase complex assembly factor 1	-0.153	-0.524	-0.248
GT_Mm_44k_51_P365521	Atp5o	ATP synthase, H+ transporting, mitochondrial F1 complex, O subunit	0.106	-0.084	-0.097
GT_Mm_44k_51_P410823	Sdha	Succinate dehydrogenase complex, subunit A, flavoprotein (Fp)	-0.639	-0.851	-2.143
GT_Mm_44k_51_P472671	Uqcrb	Ubiquinol-cytochrome c reductase binding protein	0.297	-0.117	-0.586
GT_Mm_44k_51_P478303	Cyp2d9, 12	Cytochrome P450, family 2, subfamily d, polypeptide 9	-0.606	-0.947	-0.807
GT_Mm_44k_51_P482051	Cyp3a41b, 16	Cytochrome P450, family 3, subfamily a, polypeptide 16	-0.488	-0.452	-0.942
GT_Mm_44k_51_P489367	Cyp3a25	Cytochrome P450, family 3, subfamily a, polypeptide 25	-0.986	-1.262	-2.248
GT_Mm_44k_52_P136153	Cox7c	Cytochrome c oxidase, subunit VIIc	0.123	-0.786	-0.804
GT_Mm_44k_52_P170054	Ndufv3	NADH dehydrogenase (ubiquinone) flavoprotein 3	-0.633	-0.489	-0.731
GT_Mm_44k_52_P217474	Ndufa6	NADH dehydrogenase (ubiquinone) 1 alpha subcomplex, 6 (B14)	-0.470	-0.513	-1.425
GT_Mm_44k_52_P380314	Cyp3a25	Cytochrome P450, family 3, subfamily a, polypeptide 25	-0.540	-1.036	-1.462
GT_Mm_44k_52_P423424	Ero1l	ERO1-like (S. cerevisiae)	-1.045	-0.432	-0.770
GT_Mm_44k_52_P444628	Cybb	Cytochrome b-245, beta polypeptide	-2.624	0.021	-0.841
GT_Mm_44k_52_P44914	Cyp4a12a	Cytochrome P450, family 4, subfamily a, polypeptide 12a	-2.327	-1.619	-1.530

GT_Mm_44k_52_P595871	Cyp1a2	Cytochrome P450, family 1, subfamily a, polypeptide 2	-1.452	-1.410	-2.324
GT_Mm_44k_52_P681787	Cyb561	Cytochrome b-561	-1.004	-0.584	-0.425
GT_Mm_44k_03237	Gm10291	Glyceraldehyde-3-phosphate dehydrogenase pseudogene	-1.704	-0.429	-1.079
GT_Mm_44k_51_P182828	Pgk1	Phosphoglycerate kinase 1	-0.608	-0.164	-1.312
GT_Mm_44k_51_P189442	Adh4 ; Adh6-ps1	Alcohol dehydrogenase 4 (class II), pi polypeptide	-0.809	-0.851	-1.289
GT_Mm_44k_51_P310896	Pfkl	Phosphofructokinase, liver, B-type	-0.227	-0.625	-0.264
GT_Mm_44k_51_P312755	Ins1	Insulin I	0.506	1.039	1.206
GT_Mm_44k_51_P327295	Akr1a4	Aldo-keto reductase family 1, member A4 (aldehyde reductase)	-0.464	-0.413	-1.096
GT_Mm_44k_51_P364146	Ldha	Lactate dehydrogenase A	-1.382	-0.866	-2.390
GT_Mm_44k_51_P404275	Adh5	Alcohol dehydrogenase 5 (class III), chi polypeptide	-0.824	-0.275	-1.203
GT_Mm_44k_51_P426886	Gpi1	Glucose phosphate isomerase 1	0.224	-0.356	0.641
GT_Mm_44k_51_P428555	Adh1	Alcohol dehydrogenase 1 (class I)	0.286	-0.407	-0.650
GT_Mm_44k_51_P462385	G6pc	Glucose-6-phosphatase, catalytic	0.765	-0.992	1.384
GT_Mm_44k_51_P510418	Aldh1b1	Aldehyde dehydrogenase 1 family, member B1	-1.791	-0.650	-1.758
GT_Mm_44k_52_P259537	Gck	Glucokinase	-1.039	-0.754	-1.248
GT_Mm_44k_52_P407060	Ins2	Insulin II	0.472	2.214	1.194
GT_Mm_44k_52_P568792	Pdhb	Pyruvate dehydrogenase (lipoamide) beta	-0.656	-0.537	-1.403
GT_Mm_44k_52_P748882	Eno2	Enolase 2, gamma neuronal	0.406	-0.115	1.162
GT_Mm_44k_52_P98387	Aldoa	Aldolase A, fructose-bisphosphate	-0.125	-0.361	-0.086
**Expression of fatty acid, cholesterol, steroid and triglyceride metabolism related genes**					
GT_Mm_44k_07860	Cyp4a29	Cytochrome P450, family 4, subfamily a,	0.453	0.818	1.476

		polypeptide 29			
GT_Mm_44k_51_P289742	Slc27a5	Solute carrier family 27 (fatty acid transporter), member 5	-0.416	-0.486	-1.203
GT_Mm_44k_51_P324633	Elovl3	Elongation of very long chain fatty acids (FEN1/Elo2, SUR4/Elo3, yeast)-like 3	-2.667	-2.918	-3.309
GT_Mm_44k_01454	Mut	Methylmalonyl-Coenzyme A mutase	0.893	0.136	-0.422
GT_Mm_44k_51_P153170	Cyb5r3	Cytochrome b5 reductase 3	-0.979	-0.431	-1.647
GT_Mm_44k_51_P243755	Slc10a2	Solute carrier family 10, member 2	-1.673	-1.427	0.248
GT_Mm_44k_51_P245123	Baat	Bile acid-Coenzyme A: amino acid N-acyltransferase	0.291	0.072	0.395
GT_Mm_44k_51_P255817	Scarb1	Scavenger receptor class B, member 1	-1.164	-0.885	-1.369
GT_Mm_44k_51_P278334	Vldlr	Very low density lipoprotein receptor	1.328	1.674	0.759
GT_Mm_44k_51_P329711	Idi1	Isopentenyl-diphosphate delta isomerase	0.212	0.433	-1.050
GT_Mm_44k_51_P336060	Lcat	Lecithin cholesterol acyltransferase	-1.235	-0.713	-0.986
GT_Mm_44k_51_P355943	Mvd	Mevalonate (diphospho) decarboxylase	-0.529	-0.660	-0.829
GT_Mm_44k_51_P380881	Med15	Mediator complex subunit 15	-1.354	-0.346	-0.610
GT_Mm_44k_51_P385598	Slc37a4	Solute carrier family 37 (glucose-6-phosphate transporter), member 4	1.101	-0.270	0.699
GT_Mm_44k_51_P461429	Cyp7b1	Cytochrome P450, family 7, subfamily b, polypeptide 1	-1.130	-0.410	0.225
GT_Mm_44k_51_P514449	Nr5a2	Nuclear receptor subfamily 5, group A, member 2	0.533	0.526	0.861
GT_Mm_44k_52_P150236	Apom	Apolipoprotein M	-0.369	-0.383	-0.151
GT_Mm_44k_52_P244193	Cd24a	CD24a antigen	-0.633	-0.727	-0.219
GT_Mm_44k_52_P251403	Tgfbr2	Transforming growth factor, beta receptor II	0.513	1.025	1.321
GT_Mm_44k_52_P485971	Scap	SREBF chaperone	0.379	-0.325	-0.503

GT_Mm_44k_52_P488427	Sec14l2	SEC14-like 2 (S. cerevisiae)	-1.203	-0.656	-1.260
GT_Mm_44k_52_P636752	Cyp51	Cytochrome P450, family 51	-1.426	0.852	-0.863
GT_Mm_44k_00911	Cyp2d10;11 ; 22	Cytochrome P450, family 2, subfamily d, polypeptide 22	-0.660	-1.147	-1.279
GT_Mm_44k_01406	Cyp4a14	Cytochrome P450, family 4, subfamily a, polypeptide 14	4.307	2.209	4.419
GT_Mm_44k_01692	Aste1	Asteroid homolog 1 (Drosophila)	0.926	0.055	0.450
GT_Mm_44k_51_P120295	Cyp2d26	Cytochrome P450, family 2, subfamily d, polypeptide 26	0.234	-0.419	0.446
GT_Mm_44k_51_P122170	Rdh7	Retinol dehydrogenase 7	-0.552	-0.638	-0.828
GT_Mm_44k_51_P134142	Cyp2c70	Cytochrome P450, family 2, subfamily c, polypeptide 70	-0.542	-1.009	-0.435
GT_Mm_44k_51_P140803	Slco1b2	Solute carrier organic anion transporter family, member 1b2	-0.432	-0.315	-0.645
GT_Mm_44k_51_P144581	Pgrmc1	Progesterone receptor membrane component 1	-0.256	-0.116	-1.672
GT_Mm_44k_51_P153170	Cyb5r3	Cytochrome b5 reductase 3	-0.979	-0.431	-1.647
GT_Mm_44k_51_P213476	Pgr	Progesterone receptor	0.718	1.313	1.515
GT_Mm_44k_51_P278550	Tecr	Trans-2,3-enoyl-CoA reductase	-0.455	-0.666	-1.096
GT_Mm_44k_51_P418056	Sc5d	Sterol-C5-desaturase homolog (S. cerevisae)	0.233	0.174	-0.998
GT_Mm_44k_51_P421418	Hsd3b1	Hydroxy-delta-5-steroid dehydrogenase, 3 beta- and steroid delta-isomerase 1	-1.951	-1.899	-1.176
GT_Mm_44k_51_P430973	Paqr7	Progestin and adipoQ receptor family member VII	-0.698	-0.627	-1.802
GT_Mm_44k_51_P435911	Hsd3b7	Hydroxy-delta-5-steroid dehydrogenase, 3 beta- and steroid delta-isomerase 7	-0.771	-0.791	-1.315
GT_Mm_44k_51_P445662	Hsd17b4	Hydroxysteroid (17-beta) dehydrogenase 4	-0.351	-0.175	-1.011

GT_Mm_44k_51_P478303	Cyp2d9; d12	Cytochrome P450, family 2, subfamily d, polypeptide 9	-0.606	-0.947	-0.807
GT_Mm_44k_51_P482051	Cyp3a41b ; a16	Cytochrome P450, family 3, subfamily a, polypeptide 16	-0.488	-0.452	-0.942
GT_Mm_44k_51_P489367	Cyp3a25	Cytochrome P450, family 3, subfamily a, polypeptide 25	-0.986	-1.262	-2.248
GT_Mm_44k_51_P493649	Sult1e1	Sulfotransferase family 1E, member 1	-1.252	0.405	2.340
GT_Mm_44k_51_P496162	Hsd3b5	Hydroxy-delta-5-steroid dehydrogenase, 3 beta- and steroid delta-isomerase 5	-2.942	-2.279	-1.754
GT_Mm_44k_51_P510418	Aldh1b1	Aldehyde dehydrogenase 1 family, member B1	-1.791	-0.650	-1.758
GT_Mm_44k_51_P521128	Ncoa6	Nuclear receptor coactivator 6	0.306	0.136	-0.538
GT_Mm_44k_52_P132165	Hsd17b11	Hydroxysteroid (17-beta) dehydrogenase 11	-0.682	-0.503	-1.760
GT_Mm_44k_52_P189567	Rnf4	Ring finger protein 4	0.617	-0.122	0.416
GT_Mm_44k_52_P283258	Carm1	Coactivator-associated arginine methyltransferase 1	1.098	0.285	1.552
GT_Mm_44k_52_P44914	Cyp4a12a	Cytochrome P450, family 4, subfamily a, polypeptide 12a	-2.327	-1.619	-1.530
GT_Mm_44k_52_P485971	Scap	SREBF chaperone	0.379	-0.325	-0.503
GT_Mm_44k_52_P519689	Rxrb	Retinoid X receptor beta	-0.511	-0.710	-1.144
GT_Mm_44k_52_P532559	Thrb	Thyroid hormone receptor beta	-4.380	-0.746	-1.770
GT_Mm_44k_52_P595871	Cyp1a2	Cytochrome P450, family 1, subfamily a, polypeptide 2	-1.452	-1.410	-2.324
GT_Mm_44k_52_P636752	Cyp51	Cytochrome P450, family 51	-1.426	0.852	-0.863
GT_Mm_44k_01339	Mttp	Microsomal triglyceride transfer protein	0.077	0.155	0.066
GT_Mm_44k_51_P197213	Pnpla2	Patatin-like phospholipase domain containing 2	0.627	0.404	0.959
GT_Mm_44k_51_P259	Lpl	Lipoprotein lipase	-0.938	-0.588	-1.496

296					
GT_Mm_44k_51_P290207	Insig1	Insulin induced gene 1	-0.384	-0.237	-1.236
GT_Mm_44k_52_P113250	Insig2	Insulin induced gene 2	-0.227	-0.437	1.075
GT_Mm_44k_52_P447929	Tnxb	Tenascin XB	0.489	0.911	1.206
GT_Mm_44k_52_P572808	Agpat9	1-acylglycerol-3-phosphate O-acyltransferase 9	0.156	0.506	0.987
GT_Mm_44k_52_P641185	Ptpn11	Protein tyrosine phosphatase, non-receptor type 11	-0.454	0.124	-0.515
